# Proliferative Verrucous Leukoplakia Revisited: A Retrospective Clinicopathological Study

**DOI:** 10.3390/clinpract11020048

**Published:** 2021-06-01

**Authors:** Snehashish Ghosh, Roopa S. Rao, Manoj K. Upadhyay, Karuna Kumari, D. Sharathkumar Sanketh, A. Thirumal Raj, Sameena Parveen, Yaser Ali Alhazmi, Ankur Jethlia, Shazia Mushtaq, Sachin Sarode, Rodolfo Reda, Shankargouda Patil, Luca Testarelli

**Affiliations:** 1 Department of Oral Pathology, M. B. Kedia Dental College and Teaching Hospital, Birgunj 44300, Nepal; ghoshrocks2004@gmail.com; 2 Department of Oral Pathology, Faculty of Dental Sciences, Ramaiah University of Applied Sciences, Bangalore 560054, India; drroopasrao1971@gmail.com; 3 Department of Conservative Dentistry and Endodontics, M. B. Kedia Dental College and Teaching Hospital, Birgunj 44300, Nepal; sophiabhandari118@gmail.com; 4 Private Dental Practice, Hyderabad 500081, India; karuna2202@gmail.com; 5 Private Dental Practice, Chennai 600030, India; sankethsharathop@gmail.com; 6 Department of Oral Pathology and Microbiology, Sri Venkateswara Dental College and Hospital, Chennai 600130, India; thirumalraj666@gmail.com; 7 Department of Maxillofacial Surgery and Diagnostic Sciences, College of Dentistry, Jazan University, Jazan 45142, Saudi Arabia; sparvn@gmail.com (S.P.); jethliaankur@yahoo.co.in (A.J.); 8 Department of Maxillofacial Surgery and Diagnostic Sciences, Division of Oral Pathology, College of Dentistry, Jazan University, Jazan 45142, Saudi Arabia; yalhazmi@jazanu.edu.sa (Y.A.A.); dr.ravipatil@gmail.com (S.P.); 9 Dental Health Department, College of Applied Medical Sciences, King Saud University, Riyadh 11451, Saudi Arabia; smushtaqdr@gmail.com; 10 Department of Oral Pathology and Microbiology, Dr. D.Y. Patil Dental College and Hospital, Dr. D.Y. Patil Vidyapeeth, Sant-Tukaram Nagar, Pimpri, Pune 411018, India; drsachinsarode@gmail.com; 11 Department of Oral and Maxillofacial Sciences, Sapienza University, University of Rome, 00185 Rome, Italy; luca.testarelli@uniroma1.it

**Keywords:** leukoplakia, oral disease, oral pathology, proliferative verrucous leukoplakia

## Abstract

(1) Objective: To review the criteria proposed by Cerero-Lapiedra et al. and to retrospectively identify the under-diagnosed disease in patients diagnosed with proliferative verrucous leukoplakia. (2) Materials and methods: In this study, we included patients who were diagnosed with leukoplakia (histological label consistent with the clinical diagnosis, *n* = 95), and cases with a final diagnosis within the spectrum of proliferative verrucous leukoplakia (*n* = 110) as defined by Batsakis et al. We applied the criteria proposed by Cerero-Lepiedra et al. to screen for the possible cases of proliferative verrucous leukoplakia. (3) Results: Although many of our patients satisfied specific isolated criteria, only 11 cases satisfied specific combinations of the guidelines to satisfy a diagnosis of proliferative verrucous leukoplakia. However, due to the lack of follow-up data, the disease is not confirmed in these 11 cases. (4) Conclusion: A limited number of cases of proliferative verrucous leukoplakia were diagnosed using the criteria given by Cerero-Lapiedra et al. The true natural history of the disease could not be studied due to the lack of follow-up data. (5) Clinical relevance: Proliferative verrucous leukoplakia presenting as hyperkeratosis or mild epithelial dysplasia are often not followed up, and they subsequently transform into carcinoma. Thus, clinicians must be vigilant whenever they encounter leukoplakia, especially with multifocal presentations. In such cases, the follow-up data are the key to understanding the true nature of the disease entity.

## 1. Introduction

Proliferative verrucous leukoplakia (PVL), a unique form of leukoplakia, was first reported by Hansen et al. [1] in a long-term study of 30 patients. In this study, PVL clinically commences as an isolated homogenous leukoplakia lesion, microscopically manifesting as simple hyperkeratosis without dysplasia. Over a protracted period, the lesions spread to affect other locations. These lesions tend to recur and become exophytic with or without an erythematous component. The lesions were also reported to be slow-growing, persistent, and irreversible.

Depending upon the severity of the lesion under microscope, PVL is histopathologically graded from 0 to 10. The histopathological spectrum ranges as simple hyperkeratosis, varying grades of epithelial dysplasia, verrucous hyperplasia (VH), verrucous carcinoma (VC), papillary squamous cell carcinoma (PSCC), and oral squamous cell carcinoma (OSCC) with intermediate stages. However, Batsakis et al. opined PSCC to be a distinct clinicopathological entity [2]. They did not concur with the illustrations of PSCC in the report of Hansen et al. [1] and therefore, they revised the grading of PVL into 4 histological stages. There have been many case reports, case series, and reviews ever since Hansen et al. originally described the disease [3,4,5,6,7,8,9,10,11,12,13].

The World Health Organization (WHO) describes PVL as a distinct and aggressive form of oral and potentially malignant disease. It is multifocal, which has a progressive course, and is associated with high recurrence and malignant transformation rates. [14].

Based on case series and literature reviews, Cerero-Lapiedra et al. attempted to establish a set of diagnostic criteria (Table 1) for PVL [15]. The criteria included a set of five major criteria, four minor criteria, and specific combinations of the two to diagnose the lesion. Furthermore, in 2013, Carrard et al. modified these criteria for the diagnosis of PVL [16]. García-Chías et al. recently evaluated the criteria of Cerero-Lapiedra et al. and concluded that it could be usefully implemented for an early diagnosis of PVL [17].

In 2018, Villa et al. introduced the term “Proliferative leukoplakia” instead of PVL to diagnose similar looking lesions [18]. The authors reported the criteria for diagnosis of PVL by Cerero-Lapiedra et al. [15] to be more comprehensive than the criteria used by Villa A et al. [18], which could be largely attributed to the exclusion of progression of a lesion.

In the present study, we aim to clinicopathologically review selected patient records based on the criteria described by Cerero-Lapiedra et al. [15]. We also aim to identify the possible under- or over-diagnosed PVL cases retrospectively.

## 2. Materials and Methods

We retrospectively selected patients diagnosed between 2015 and 2019 from the archives of Oral Pathology laboratories in India. The inclusion criteria are listed below:

(1) Patients with a provisional diagnosis of leukoplakia, and their corresponding slides. Cases with any histopathological label with the clinical diagnosis of leukoplakia (*n* = 95) were included in the study (group 1). Any histopathological diagnosed cases without the available clinical information were excluded from the study.
(2) A diagnosis of PVL is usually made retrospectively, since it represents a disease that progresses in a continuum over time. There are chances that the patient may have PVL in any one of the transitional stages of the continuum. Hence, we also extracted archival slides of other oral lesions (*n* = 110), with a histological diagnosis within the spectrum of PVL (group 2), described by Batsakis et al. [2]; i.e., VH, VC, and OSCC (Table 2).

In total, we had 205 cases with 25 cases of hyperkeratosis, 83 cases of epithelial dysplasia (mild—46, moderate—25, severe—12), 11 cases of VH, 9 cases of VC, and 77 cases of OSCC. The OSCC cases consisted of 52 well-differentiated OSCC (WDSCC), 22 moderately differentiated OSCC (MDSCC), and 3 poorly differentiated OSCC (PDSCC). All corresponding slides were reviewed and reconfirmed. Patient information recorded in the pathology request forms including the patients’ age, gender, habits, and other clinical details of the lesions were extracted, including description and size of lesion, number of sites involved, provisional and final diagnosis (Table 3).

Once the clinical and histopathological data were obtained and reconfirmed, the criteria for PVL proposed by Cerero-Lapiedra et al. [15] (Table 1) were applied to these reviewed cases.

According to the guidelines, a case of PVL must satisfy either 3 major criteria (MC) or 2 major criteria (MC) and 2 minor criteria (mc) with the histopathology being a mandatory criterion in either combination.

**Figure 1:**
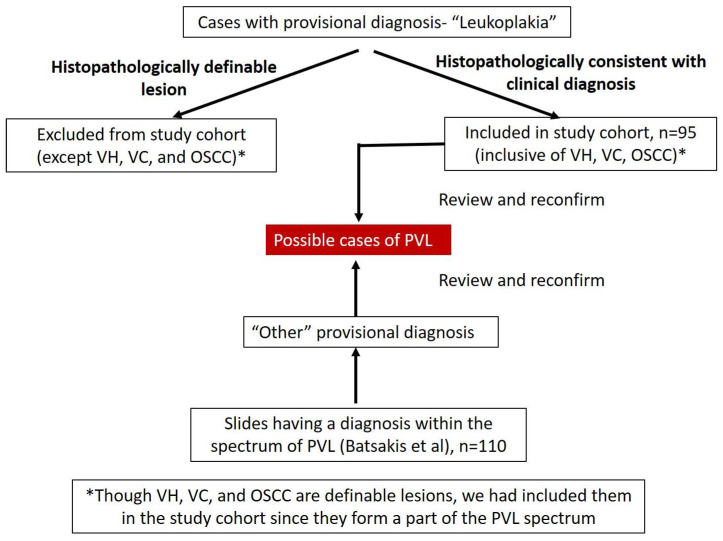
Summary of the methodology employed in the selection of cases.

Exclusion criteria: Post clinical diagnosis of oral leukoplakia, if the lesion does not fall in to the PVL histopathological spectrum and is clearly histopathologically defined as any lesion (example Oral lichen planus, oral submucous fibrosis etc.) other than OVC, OVH, and OSCC, we excluded them from the present study. Figure 1 summarizes the methodology employed in the selection of cases.

**Figure 2:**
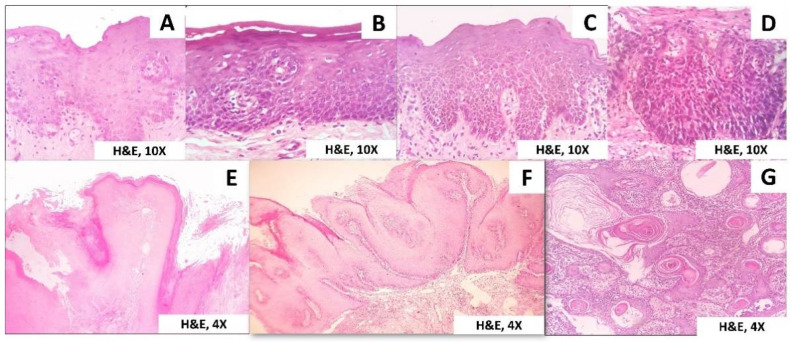
Histopathological spectrum of PVL. (**A**) Mild epithelial dysplasia, (**B**) Moderate epithelial dysplasia, (**C**) Severe epithelial dysplasia, (**D**) Carcinomas in situ, (**E**) Verrucous hyperplasia, (**F**) Verrucous carcinoma, (**G**) Squamous cell carcinoma.

The histopathological spectrum of PVL includes simple hyperkeratosis, varying grades of epithelial dysplasia, verrucous hyperplasia (VH), verrucous carcinoma (VC), papillary squamous cell carcinoma (PSCC), and oral squamous cell carcinoma (OSCC) with intermediate stages (Figure 2).

## 3. Results

We collected the clinicopathologic data, by including cases of leukoplakia (*n* = 95) that had a final diagnosis consistent with the provisional diagnosis (inclusive of VH, VC, and OSCC) (group 1) and retrieving histopathology slides of lesions with a diagnosis within the spectrum of PVL (group 2) proposed by Batsakis et al. (*n* = 110). All data (both clinical and histopathological) were tabulated (Table 3). A total of 205 lesions were screened, with the age range being 19–85 years. There were 141 males and 64 females with a ratio of 2.2:1. In our cohort, we observed 86.3% patients were having the habits of smoking, smokeless tobacco, betel quid, and alcohol.

After applying the guidelines (Cerero-Lapiedra et al.) to the data obtained, we found 7.3%, 14%, 0.9%, and 0.9% of the cases were positive for MC: “A”, “B”, “C”, and “D” respectively (Table 4).

All cases were positive for the criterion “E”, since it was the initial inclusion criterion for our study. As for the mc, 22.9%, 31.2%, and 13.6% were positive for the criterion “a”, “b”, and “c”, respectively. Unfortunately, we could not obtain the follow-up/evolution data of the lesions in our study, and hence, no lesion satisfied criterion “d”.

All though many lesions could satisfy the specific isolated criterion, only 5.3% (*n* = 11) of the patients in our series satisfied the combinations of the guidelines to fully satisfy a diagnosis of PVL (Table 4 and Table 5). Table 5 shows a summary of lesions that satisfied specific combinations for the diagnosis of PVL along with their demographic and clinical characteristics. Figure 2 summarizes the representative pictures of the spectrum of cases noted in PVL.

Cases satisfying the PVL diagnosis: As summarized in Table 5, among group 1 lesions, 5.3% (*n* = 5) were diagnosed as PVL. Group 2 lesions had 5.4% (*n* = 6) satisfying the diagnosis of PVL. In total, 5.3% (11/205) of the total number of patients were given a diagnosis of PVL. The mean age of the patients with PVL was 56.36.

We found 63.6% (7/11) of the cases to be female and the rest (36.4%, 4/11) to be male.

Furthermore, it is important to note that 11% (7/64) of the total female patients selected were diagnosed with the disease in contrast to 2.8% (4/141) of the total males.

Habits were harbored by 63.6% of patients.

In group 1, 3/5 cases had a microscopic diagnosis of OSCC, two of which were WDSCC, and one was MDSCC. The histopathological diagnoses of the other two lesions in group 1 were moderate epithelial dysplasia and hyperkeratosis without dysplasia. Group 2 had five lesions with a microscopic diagnosis of OSCC and one with a diagnosis of VH. Among the five OSCCs, 3/5 were WDSCCs, 1/5 was MDSCC, and one was diagnosed as micro-invasive OSCC.

The most affected site was buccal mucosa (8/11) followed by retro-molar trigone (4/11), lip and alveolar ridge (3/11 each), lateral tongue, and gingiva (1/11 each). We found the most common MC to be “A” followed by “B” and “D”. Similarly, the most prevalent mc was “a” and “b” followed by “c”. Data for minor criterion “d” were lacking. All cases were positive for criterion “E”.

Although these 11 lesions satisfied the guidelines set by Cerero-Lapiedra et al. [15], we opine that the lack of data on evolution, recurrence, and follow-up of the lesions as a major reason for the scepticism of the diagnoses of these 11 patients. Hansen et al. mentioned that PVL was a lesion that progressed/evolved. It has to be remembered that not all multi-focal leukoplakic lesions can be considered to be PVL, taking into consideration the field cancerization phenomenon [19,20,21].

## 4. Discussion

The diagnosis of PVL remains an enigma ever since its first report by Hansen et al. [1]. PVL progresses from an isolated leukoplakia to become multi-focal confluent or isolated exophytic/verrucous lesions.

The incidence of PVL is mostly reported in elderly women, with a mean age of 70 years. Patients may or may not have tobacco habits and often have a history of a long-time awareness of a leukoplakic lesion, sometimes for more than two decades [22,23]. Zakrzewska et al. reported PVL as a distinct clinicopathological entity in a patient-based study on London population [3]. They proposed a long-term follow-up with regular reviews, and careful examination of the oral cavity is required for the identification of new lesions.

Silverman and Gorsky, in their follow-up study of 54 PVL cases, attributed the failure of multiple therapies in eliminating the lesions to unidentifiable subcellular changes leading to the recurrence of disease [4].

The high rate of malignant transformation (60% to 100%) of PVL signifies an early and accurate diagnosis of the lesion [7].

Cabay et al. reiterated the importance of a sub-epithelial inflammatory infiltrate in the superficial stroma, which may be intense and obscure the basement membrane similar to oral lichen planus [7]. They hypothesized cases of leukoplakia showing lichenoid interface inflammation with basal cell hyperplasia and hyperkeratosis without evidence of basilar vasculopathy as a potential stage of PVL.

Ghazali et al. carried out a retrospective analysis of nine multifocal verrucous lesions retrieved from their surgical and histopathological records [5]. Using the criteria of Hansen et al., they attempted to describe the clinicopathological features of these nine lesions to identify possible cases of PVL. However, no case satisfied the criteria used, albeit three cases were indicative of PVL.

Villa et al. [18] proposed the term “proliferative leukoplakia” instead of PVL, because the presence of a verrucous area was not present in many of the cases satisfying the other criteria of PVL. Their criteria for diagnosing the PVL was a modified version of Cerero-Lapiedra et al. [15].

Favia et al. followed up forty-eight cases of PVL over a period ranging from eighteen months to two hundred and forty months to observe the malignant transformation and reported that fifteen out of forty-eight cases developed only one primary tumor, whereas the remaining thirty-three cases developed two or more primary tumors [24]. Upadhyaya et al. [25] conducted a similar study in 2018 to find out the malignant potential of PVL and its possible association with Human Papilloma Virus (HPV). The authors reported malignant transformation of the lesion in 50% of the observed cases with no significant association with HPV infection.

Cerero-Lapiedra et al. proposed a set of guidelines in 2010 to establish an objective and early diagnosis of PVL [15]. They proposed that the early detection of PVL is adequate for the management of lesion. For the identification of PVL, they put forward that the lesion should fulfill certain specific combinations of criteria. The criteria consisted of five major and four minor criteria, of which a lesion should satisfy either three major or two major and two minor criteria. These guidelines have been recently verified and evaluated by García-Chías and colleagues [17]. They concur that the guidelines allow clinicians to make an early diagnosis of PVL so that 60% of the patients with the diagnosis end up with the disease.

Our retrospective study was an attempt to possibly discern misdiagnosed/underdiagnosed cases of PVL. We used the guidelines recommended by Cerero-Lapiedra et al. for the same [15]. We included two groups of lesions: one from clinical and the other from histological perspective.

We furnished a tentative diagnosis of PVL for 11 lesions from a total of 205 lesions included. We found the most common major criterion to be [A] and [E] and the most frequent minor criterion to be [a] and [b]. These findings were similar to García-Chías et al., who reported minor criterion [b] and [c] to be most frequent [17].

However, our clinical records lacked information on the follow-up of patients, recurrence, and evolution of the lesions. These limitations must be taken into consideration when rendering a diagnosis of PVL.

## 5. Conclusions

PVL is a high-risk disease with a high rate of malignant transformation that must be detected early for management. Diagnostic criteria for a disease are an important aid, and it is critical to observe and check if the patient fulfills them. However, we opine that a very strict and rigid application of a set of criteria to any disease process may often result in under-diagnosis of the condition. On the application of Cerero-Lapiedra’s criteria, we found 11 cases to be positive for a diagnosis of PVL.

However, we believe the diagnoses of PVL in the present study to be uncertain due to insufficient data on the follow-up of patients and the evolution of the disease. Thus, clinicians must ensure that they follow-up multi-focal oral leukoplakia, irrespective of the inert nature of their initial histopathology. Only thorough follow-up data would provide adequate evidence for a conclusive PVL diagnosis.

## Figures and Tables

**Table 1:** Proposed criteria for proliferative verrucous leukoplakia (Cerero-Lepiedra et al. [15]).

Major Criteria (MC)	Minor Criteria (mc)
A leukoplakia lesion with more than two different oral sites (A)	Leukoplakia lesion occupies at least 3 cm when adding all the affected areas (a)
Existence of a verrucous area (B)	Patient is female (b)
Lesions have spread or engrossed during the course of the disease (C)	Patient (male or female) is a non-smoker (c)
Recurrence in a previously treated area (D)	Disease evolution higher than 5 years (d)
Histopathology diagnosis (E *)	

E *—The histopathological diagnosis consistent with the clinical diagnosis of leukoplakia under the spectrum of PVL, i.e., hyperkeratosis, epithelial dysplasia, verrucous carcinoma, verrucous hyperplasia, squamous cell carcinoma.

**Table 2:** Total number of cases retrieved.

Provisional Diagnosis	Final Diagnosis							
	Hyperkeratosis	Dysplasia			VH	VC	OSCC	Total
		M	Mo	S				
**Leukoplakia**	18	40	20	02	05	02	08	95
Others *	7	6	5	10	6	7	69	110
**Total**	25	46	25	12	11	9	77	205

Others *—Any lesions without the provisional diagnosis of leukoplakia, but with the histopathological diagnosis consistent with the spectrum of PVL. VH—verrucous hyperplasia, VC—verrucous carcinoma, OSCC—oral squamous cell carcinoma, M—mild, Mo—moderate, S—severe.

**Table 3:** Demographic and clinical characteristics of the study.

Lesions		Hyperkeratosis	Epithelial Dysplasia			VC	VH	OSCC		
		*n* = 25	*n* = 83			*n* = 9	*n* = 11	*n* = 77		
			M *n* = 46	Mo *n* = 25	S *n* = 12			WD *n* = 52	MD *n* = 22	PD *n* = 3
Age	Mean	47.4	45	49.2	56	64.7	54.45	56.44	53.8	47.66
	SD	16.317	14.995	14.957	10.807	9.022	17.403	12.475	13.757	24.00
	Range	20–71	19–75	28–79	41–72	46–78	28–85	28–83	29–75	18–65
Gender	Male	17	40	23	8	6	9	23	13	2
	Female	8	6	2	4	3	2	29	9	1
Site of lesion	Buccal mucosa	20	37	17	8	4	7	28	5	1
	Gingiva	4	0	2	1	1	1	2	0	1
	Labial mucosa	2	2	0	1	0	0	0	0	0
	Gingival buccal Sulcus	0	0	0	0	2	3	5	1	0
	Retro molar Trigone	4	6	5	0	2	1	2	3	0
	Lower Lip	2	3	1	0	0	2	3	0	0
	Tongue	1	1	2	2	0	2	4	7	0
	Alveolar ridge	2	4	2	2	2	1	10	7	1
	Palate	0	1	0	0	0	1	3	1	1
Description	Patch	22	35	20	6	1	4	6	1	0
	Exophytic (Plaque/Verruco- Papillary	3	9	1	1	5	2	5	2	2
	Ulcer	0	2	2	2	0	0	19	6	1
	Ulcero-proliferative	0	0	2	3	3	5	22	13	0
Colour	White	20	40	15	8	3	7	18	4	0
	Red/pink	0	0	1	3	0	1	7	5	1
	Mixed (Red-White)	5	6	9	1	6	3	27	13	2
Size(cm)	<1	2	3	1	2	0	1	5	1	0
	1–2	20	18	13	3	1	4	25	9	3
	2–4	0	19	10	6	7	0	18	12	0
	>4	3	6	1	1	1	6	4	0	0
Habits	Smokeless tobacco	10	16	10	9	7	5	41	13	2
	Smoking	6	22	17	6	2	4	20	11	2
	Betel quid	2	3	0	2	0	1	2	0	0
	Alcohol	0	0	0	0	0	0	2	0	0
	No Habits	10	7	1	1	2	2	3	1	1

M—mild, Mo—moderate, S—severe, WD—well-differentiated, MD—moderately differentiated, PD—poorly differentiated.

**Table 4:** Patients with positive criteria.

Major Criteria (mc)	No. of Cases with Positive Criteria *n* (%)	Minor Criteria (mc)	No. of Cases with Positive Criteria *n* (%)
A leukoplakia lesion with more than two different oral sites (A)	15 (7.3%)	Leukoplakia lesion occupies at least 3 cm when adding all the affected areas (a)	47 (22.9%)
Existence of a verrucous area (B)	30 (14%)	The patient is female (b)	64 (31.2%)
Lesions have spread or engrossed during the course of the disease (C)	2 (0.9%)	Patient (male or female) is a non-smoker (c)	28 (13.6%)
Recurrence in a previously treated area (D)	2 (0.9%)	Disease evolution higher than 5 years (d)	0
Histopathology diagnosis (E)	205 (100%)		
Total number of patients satisfying criteria for PVL–11			

**Table 5:** Summary of the 11 patients who met the diagnostic criteria proposed by Cerero-Lepiedra et al. [15].

	Case	Age	Sex	Site *	Habit **	Provisional Diagnosis	Microscopic Diagnosis	Disease Evolution (No. of Years)	MC/mc Combination
**Group 1**	1	56	F	B (L and R), P	ST	Leukoplakia	Well Diff SCC	NA	AE/ab
	2	71	M	B (L and R), RMT	S, ST	Leukoplakia	Hyperkeratosis without dysplasia	NA	ABE/a
	3	60	F	B, AR, RMT	ST	Leukoplakia	Well Diff SCC	NA	AE/ab
	4	65	F	L, TL	ST	Leukoplakia	Mod. Diff SCC	NA	AE/ab
	5	42	F	B (R and L), RMT	NO HABITS	Leukoplakia	Mod. Epithelial Dysplasia	NA	ABE/abc
**Group 2**	6	40	F	B (R & L)	NO HABITS	Verrucous Leukoplakia	Early Invasive SCC	NA	BE/bc
	7	45	F	B, AR	NO HABITS	Carcinoma	Well Diff SCC	NA	BE/bc
	8	59	M	AR, TL, TD	S	Carcinoma	Mod. Diff SCC	NA	BDE/a
	9	46	M	RMT, B, G	ST	Verrucous Leukoplakia	Microinvasive SCC	NA	ABE/a
	10	66	F	L	NO HABITS	Carcinoma	Well Diff SCC	NA	DE/bc
	11	70	M	B (R and L), L	S, ST	Verrucous Leukoplakia	Verrucous Hyperplasia	NA	ABE/a
**PVL**	**Total**		**Age (Mean)**			**Sex**		**Habits** ***n* (%)**	
						**Male** ***n* (%)**	**Female** ***n* (%)**		
	11		56.36			7 (63.6 %)	4 (36.4 %)	7 (63.63%)	

* B—Buccal Mucosa, G—Gingiva, RMT—Retromolar trigone, TD—Tongue dorsum, TL—Tongue, lateral, L—lip, AR—Alveolar ridge, P—Palate; ** S—Smoking, ST—smokeless tobacco, BQ—Betel quid, A—Alcohol, MC—Major criteria, mc—Minor criteria.

## Data Availability

No additional data was generated in the study.
